# Upregulation of Klotho Aggravates Insulin Resistance in Gestational Diabetes Mellitus Trophoblast Cells

**DOI:** 10.1155/2022/1500768

**Published:** 2022-10-21

**Authors:** Li Lin, Xinyu Wang, Weihua Zhao, Yaxuan Chen

**Affiliations:** Department of Obstetrics, Shenzhen Second People's Hospital, The First Affiliated Hospital of Shenzhen University Health Science Center, Shenzhen, Guangdong, China

## Abstract

**Objective:**

Insulin resistance (IR) plays a key role in gestational diabetes mellitus (GDM) pathogenesis. The antiaging protein klotho has been proven to be closely related to IR. The purpose of this study was to investigate the effect of klotho on IR in GDM trophoblast cells.

**Methods:**

The GDM cell model of HTR-8/SVneo cells was induced by high glucose (HG). Plasmid transfection was used to mediate the overexpression or silencing of klotho. The effects of klotho on cell viability, IR, and the IGF-1/PI3K pathways were observed by RT-qPCR, western blot, Cell Counting Kit-8 detection, glucose uptake assay, and immunofluorescence detection.

**Results:**

Klotho expression was up-regulated in HG-induced cells. Overexpression of klotho could reduce the cell viability, insulin signaling molecules (INSR-*α*, INSR-*β*, IRS1, IRS2, and GLUT4), and glucose uptake in HTR-8/SVneo cells of the HG group. In addition, the overexpression of klotho inhibited the levels of IGF-1, IGF-1R/p-IGF-1R, and the phosphorylation and activation of the signal transduction molecules PI3K/Akt/mTOR. On the contrary, klotho deletions could reverse these changes of HTR-8/SVneo cells induced by HG*. Conclusion*. In a word, the results of this study showed that the regulation of klotho played an important role in the IR of trophoblast cells induced by HG, which was mediated at least in part by the IGF-1/PI3K/Akt/mTOR pathway.

## 1. Introduction

Gestational diabetes mellitus (GDM) is one of the most common complications of pregnancy, which refers to the abnormal glucose tolerance that occurs during pregnancy or is first discovered [1]. GDM often leads to adverse pregnancy outcomes, among which macrosomia is one of the common adverse outcomes [2]. The increased risk of macrosomia in GDM is mainly due to maternal insulin resistance (IR) [3]. IR is an important factor in the pathophysiology of GDM [4]. Clinical experiments show that obese women who have reached the target for blood glucose control can get the same pregnancy outcome as normal-weight and overweight women only when they receive insulin therapy [5]. As the boundary between mother and fetus, the placenta plays a vital role in the occurrence and development of GDM. It is reported that placental insulin signals changes in obese or diabetic pregnant women that are significantly related to fetal overgrowth [6]. Presently, the regulatory mechanism of placental insulin signals in patients with GDM is unclear.

The intracellular signal of placental insulin is mediated by stimulating its tyrosine kinase receptor and activating insulin receptor substrate -1 (IRS-1) [7]. Activation of the insulin receptor can lead to tyrosine phosphorylation of insulin receptor substrates (IRS-1 and IRS-2). However, IRS proteins can also be activated by insulin-like growth factor (IGF) receptors besides insulin receptors [8, 9], thus providing a complete platform for insulin and IGF signal transmission. IGF-1 can increase glucose intake, decrease insulin secretion, and enhance insulin sensitivity [10]. Furthermore, the tyrosine phosphorylation site on the IRS-1 protein binds to various signal transduction molecules, such as phosphoinositide 3-kinase (PI3K). PI3K activation leads to protein kinase B (Akt) signal transduction, which is usually called the “metabolic pathway” of insulin signaling due to the downstream effects of Akt and mammalian target of rapamycin (mTOR) signal targets on glucose, lipid, and protein metabolism [11–13].

Klotho is an “antiaging” gene, which will accelerate aging when destroyed and prolong life when overexpressed [14]. Klotho is also considered an antiaging factor that induces IR and participates in the pathogenesis of diabetes [15, 16]. It has also been reported that the expression of *β*-klotho increased in the GDM placenta, which could be one of the reasons for IR [17]. In addition, Shao et al. also found that klotho expression was significantly upregulated in the placenta in macrosomia [18]. However, knocking out klotho in Lep (ob/ob) mice with leptin deficiency can reduce obesity and increase insulin sensitivity, thus lowering blood sugar levels [19]. Previous studies have shown that klotho induces down-regulation of the insulin/IGF signal [20]. In addition, klotho can down-regulate the activation of IGF-1 on IGF-1R and Akt [21].

Although klotho can regulate insulin signals to some extent, it has not been studied whether klotho can regulate placental IR in GDM. Therefore, we used HG to induce the GDM model of trophoblast cells to study the effect of klotho on the IR of GDM trophoblast cells.

## 2. Materials and Methods

### 2.1. Cell Culture

Human chorionic trophoblast cell line HTR-8/SVneo was purchased from Honorgene (Changsha, China) and cultured in RPMI-1640 medium containing 5% fetal bovine serum (FBS) at 37°C and 5% CO2. The NC-group cells were cultured in a medium with a glucose concentration of 5 mmol/L. The high-glucose (HG) group cells were cultured in the medium with a glucose concentration of 25 mmol/L. After 24 hours of treatment, cells were collected for subsequent detection.

### 2.2. Cell Transfection

Transient transfection was performed with Lipofectamine 2000 (Invitrogen, USA). A klotho silence (si-klotho group), klotho overexpression (oe-klotho group), and their corresponding negative control (Vect group) plasmids were purchased from GenePames (Shanghai, China) and transfected into the HG-induced HTR-8/SVneo, respectively. The experiments were grouped as follows: the NC group, the HG group, the HG + Vect group, the HG + si-klotho group, and the HG + oe-klotho group.

### 2.3. Quantitative Reverse Transcription PCR (RT-qPCR)

Total RNA was isolated from HTR-8/SVneo cells with Trizol reagent (Invitrogen, USA). Total RNA was used as a template and reverse transcribed into cDNA. RT-qPCR was performed with an UltraSYBR Mixture (CW2601, cwbio). The primer sequence used is shown in Table 1. Use the 2^−ΔΔCt^ method, with *β*-actin used as a reference to assess the relative level of klotho.

### 2.4. Western Blot

Total protein was extracted from cells with RIPA lysis buffer (AWB0136a, Abiowell, China). Then, the protein was separated by 10% sodium dodecyl sulfate-polyacrylamide gel electrophoresis and transferred to a nitrocellulose membrane. The membrane was sealed with 5% skim milk for 2 hours and then incubated with the primary antibody overnight at 4°C. Anti-klotho antibody (28100-1-AP, proteintech), anti-Insulin receptor-alpha (INSR-*α*) antibody (ab203037, Abcam), Insulin receptor-beta (INSR-*β*) antibody (ab69508, Abcam), anti-Glucose transporter type 4 (GLUT4) antibody (66846-1-Ig, proteintech), anti-IRS1 antibody (17509-1-AP, proteintech), anti-IRS2 antibody (20702-1-AP, proteintech), anti-IGF-1 antibody (28530-1-AP, proteintech), anti-p-IGF-1R antibody (ab5681, Abcam), anti-IGF-1R antibody (20254-1-AP, proteintech), anti-p-mTOR antibody (67778-1-Ig, proteintech) anti-mTOR (66888-1-Ig, proteintech), anti-PI3K (ab140307, Abcam), anti-p-PI3K antibody (ab182651, Abcam), anti-Akt antibody (10176-2-AP, proteintech), anti-p-Akt (66444-1-Ig, proteintech) antibody and anti-*β*-actin antibody (66009-1-Ig, proteintech) were a primary antibody. HRP-conjugated secondary anti-rabbit immunoglobulin G (IgG) antibody or anti-mouse IgG antibody was incubated for 1.5 hours. Protein blots were measured with an enhanced chemiluminescence (ECL) kit (K-12045-D50, advansta). *β*-actin was used as an internal reference.

### 2.5. Cell Counting Kit-8 (CCK-8) Detection

CCK-8 (AWC0114a, Abiowell, China) was used to evaluate cell viability. Simply put, 100 *μ*L of cells were inoculated on a 96-well plate with a cell density of 1 × 104. Then, the cells were incubated with 10 *μ*L of CCK-8 solution and set for 4 hours at 37°C. The optical density (OD) value at 450 nm was measured with a Bio-Tek microplate reader (MB-530, HEALES).

### 2.6. Glucose Uptake Assay

According to the manufacturer instructions, the glucose uptake of HTR-8/SVneo cells was measured by a fluorescent glucose analog, 2-[N-(7-nitrobenz-2-oxa-1,3-diazol-4-yl) amino]-2-deoxy-glucose (2-NBDG) (Invitrogen, N13195). Briefly, the cells were cultured in a medium with a 100 *μ*M 2-NBDG for 1 hour at 37°C. The cells were washed twice with PBS, digested with trypsin, and centrifuged for 5 min at 1500 rpm at 4°C. Then, the cells were suspended in 200 *μ*l of PBS in each tube and immediately analyzed and quantified by flow cytometry (A00-1-1102, Beckman, USA).

### 2.7. Immunofluorescence Detection

The expression and distribution of p-IGF-1R were detected by immunofluorescence staining. Cells were pre-seeded on the slide and fixed with 4% paraformaldehyde for 15 minutes. After scouring with PBS to remove residual paraformaldehyde, the cells were infiltrated with 0.1% TritonX-100 for 30 minutes to permeate the cell membrane. Then, goat serum was used to block the nonspecific antigen for 15 minutes and spent the night with the anti-p-IGF-1R antibody (ab39398, Abcam) at 4°C. After washing with PBS, the cells were incubated with secondary bodies anti-Rabbit IgG (H + L) for 90 minutes. The nucleus was incubated with DAPI. Finally, the cells were sealed with buffered glycerin and stored away from light for observation under a fluorescence microscope (Motic, Germany).

### 2.8. Data Statistics

The statistical analysis was performed by using the GraphPad Prism 8 software. All data were represented as the mean ± SD. 'Student's *t*-tests were used for the statistical analysis between the two groups, and one-way analysis of variance (ANOVA) determined three or more groups. *p* < 0.05 was considered significant.

## 3. Results

### 3.1. Klotho was Highly Expressed in HTR-8/SVneo Cells Induced by HG

**Figure 1:**
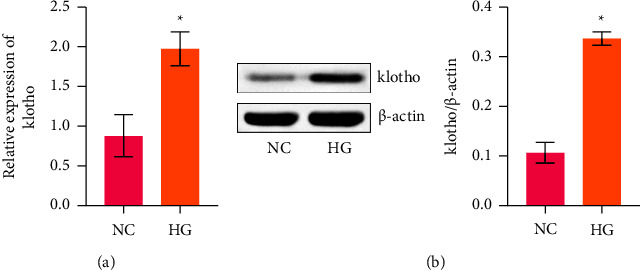
Klotho was highly expressed in HTR-8/SVneo cells induced by HG. (a) RT-qPCR of klotho levels. (b) Western blot of klotho levels. ^*∗*^*p* < 0.05 compared with the NC group.

To study the important influence of klotho on GDM trophoblast cells, we simulated the HTR-8/SVneo cell model by HG induction. RT-qPCR was performed to test klotho levels in HTR-8/SVneo cells (Figure 1(a)). The results showed that the expression of klotho in the HG group was significantly higher than that in the NC group. Next, we detected the protein level of klotho in HTR-8/SVneo cells. Western blot results identified that the klotho protein level increased significantly in the HG group compared with the NC group (Figure 1(b)). These results suggested that klotho might be participating in the occurrence and development of GDM.

### 3.2. HG Could Induce IR in HTR-8/SVneo Cells

**Figure 2:**
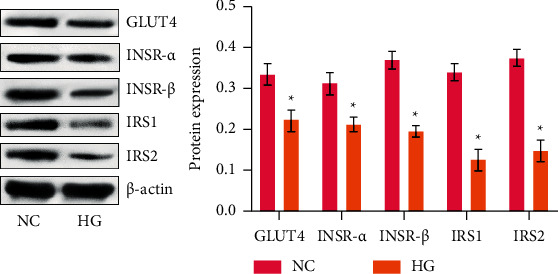
HG could induce IR in HTR-8/SVneo cells. GLUT4, INSR-*α*, INSR-*β*, IRS1, and IRS2 protein levels were profiled by western blot. ^*∗*^*p* < 0.05 compared with the NC group.

To investigate the insulin-mediated glucose utilization ability of HTR-8/SVneo cells, we detected the protein levels of the insulin signaling molecules GLUT4, INSR-*α*, INSR-*β*, IRS1, and IRS2. The results showed that GLUT4, INSR-*α*, INSR-*β*, IRS1, and IRS2 levels in trophoblast cells under HG significantly decreased than in the control group (Figure 2). This outcome indicated that trophoblast cells cultivated by HG were in IR.

### 3.3. Klotho Affected the Insulin Sensitivity of HTR-8/SVneo Cells Induced by HG

**Figure 3:**
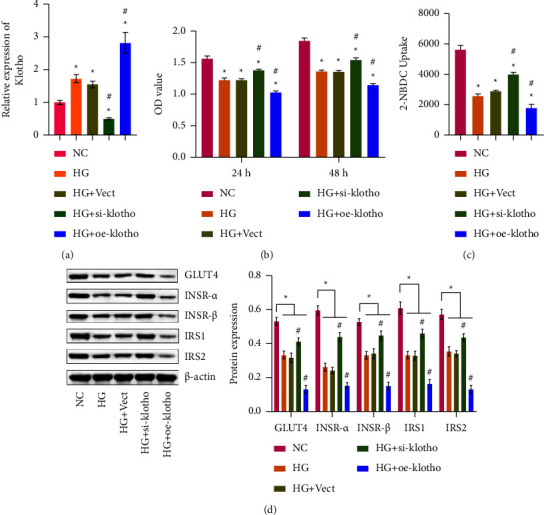
Klotho affected insulin sensitivity. (a). RT-qPCR of klotho levels. (b) CCK-8 detection was used to detect cell viability. (c) The cells were subjected to a glucose uptake assay. (d) Western blot of GLUT4, INSR-*α*, INSR-*β*, IRS1, and IRS2 protein levels. ^*∗*^*p* < 0.05 compared with the NC group. ^#^*p* < 0.05 compared with the HG group.

It has been proven that klotho is closely related to the insulin signaling pathway of cells [22]. This study further verified whether klotho damages the insulin signaling pathway of trophoblast cells caused by HG. The RT-qPCR results showed that klotho was effectively silenced or overexpressed (Figure 3(a)). CCK-8 detection showed that cell viability decreased after HG treatment compared to the NC group. Compared with the HG group, cell viability increased after klotho silence but decreased after klotho overexpression (Figure 3(b)). As shown in Figure 3(c), the glucose uptake in HTR-8/SVneo cells of the HG group was significantly decreased compared with the control group. After klotho silence, glucose uptake increased remarkably in HTR-8/SVneo cells of the HG group but decreased after klotho overexpression. Next, we detected the insulin signaling molecule levels. The results showed that compared with the HG group, the expression levels of GLUT4, INSR-*α*, INSR-*β*, IRS1, and IRS2 increased after Klotho was silenced. In contrast, the expression of these proteins was reversed after Klotho overexpression (Figure 3(d)). These results suggested that klotho might promote HG-induced HTR-8/SVneo cell IR by regulating the molecular changes of the insulin signaling pathway.

### 3.4. Klotho Affected IGF-1/PI3K Signaling Pathway

**Figure 4:**
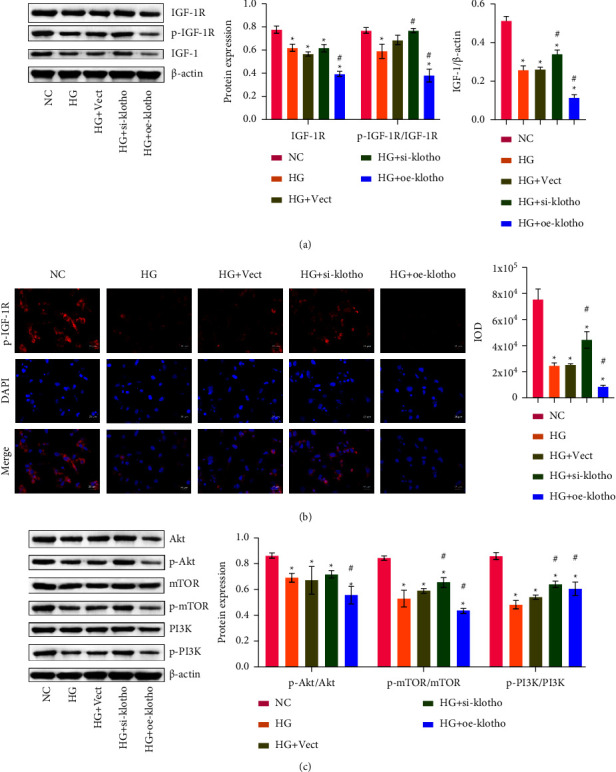
Klotho affected the IGF-1/PI3K signaling pathway. (a) IGF-1R, p-IGF-1R, and IGF-1 protein levels were tested by western blot. (b) Immunofluorescence was used to detect p-IGF-1R levels. (c) Western blot of PI3K/p-PI3K, Akt/p-Akt, and mTOR/p-mTOR protein levels. ^*∗*^*p* < 0.05 compared with the NC group. ^#^*p* < 0.05 compared with the HG group.

To further explore the influence of klotho on IR, we detected the related indexes of the IGF-1 pathway. Western blot results showed that, compared with the HG group, the levels of IGF-1R, p-IGF-1R, and IGF-1 in the HG + si-klotho group increased but decreased in the HG + oe-klotho group (Figure 4(a)). Immunofluorescence detected the expression of p-IGF-1R, and the results were consistent with western blot, suggesting that klotho might regulate the phosphorylation of IGF-1R in HTR-8/SVneo cells (Figure 4(b)). Next, we detected the expression of PI3K/p-PI3K, Akt/p-Akt, and mTOR/p-mTOR (Figure 4(c)). Compared with the HG group, the PI3K/p-PI3K, Akt/p-Akt, and mTOR/p-mTOR levels increased in the HG + si-klotho group and decreased in the HG + oe-klotho group. The above results suggested that klotho might aggravate HG-induced IR in HTR-8/SVneo cells by inhibiting the activation of the IGF-1/PI3K/Akt/mTOR signaling pathway.

## 4. Discussion

GDM often leads to placental dysplasia [23]. Trophoblast cells were an important part of the placenta, which was vital to the formation of the placenta and the development of normal fetuses [24]. HG was a characteristic of GDM, which led to trophoblast dysfunction, thus inhibiting the normal development of the placenta [25]. Therefore, we used HTR-8/SVneo cells induced by HG to study trophoblast cells' biological function and molecular changes during GDM pathology. The results showed that HG significantly inhibited the viability of HTR-8/SVneo cells, which was consistent with previous research results [26]. In addition, we also found that klotho was highly expressed in HTR-8/SVneo cells induced by HG. A down-regulation of klotho could reduce the cell viability and IR induced by HG by promoting the activation of the IGF-1, IGF-1R, and PI3K pathways. It indicated that klotho might be involved in developing diabetes during pregnancy and might be an important marker of GDM.

Many studies showed that IR played an important role in GDM [6, 27]. At the molecular level, IR is usually caused by failure of insulin signal transmission, which leads to insufficient plasma membrane translocation of GLUT4 [28]. Our study found that glucose uptake and insulin signaling molecules (INSR-*α*, INSR-*β*, IRS1, IRS2, and GLUT4) were significantly decreased in HG-induced HTR-8/SVneo cells, which is consistent with previous studies [29]. Moreover, Zhao et al. showed that the glucose transportation mediator GLUT4 was reduced in HG cultured trophoblasts [30], which was largely consistent with our study. The above results indicated that treating trophoblasts with HG medium in vitro confirms IR in the GDM placenta.

Studies have shown that klotho-mediated insulin metabolism and aging are related to IR [15, 31, 32]. The expression of klotho in the placenta of GDM-pregnant women was significantly up-regulated [17]. In our study, we found that klotho was highly expressed in HTR-8/SVneo cells induced by HG. After klotho was silenced, the cell viability, glucose uptake, and insulin signal molecules were significantly increased in HG-induced HTR-8/SVneo cells, and after klotho was overexpressed, these results were reversed. Therefore, it was speculated that klotho might participate in the insulin signaling pathway and IR and further participate in the pathological changes of GDM trophoblast cells.

It was reported that klotho inhibits the IGF signaling pathway, induces SOD expression to reduce oxidative stress, and inhibits the Akt-mTOR signaling pathway to inhibit abnormal growth of the kidney [33]. Klotho inhibited IGF signal transduction, maintained glomerular Cx 40 level, and improved albuminuria in Higa mice. Adding klotho protein could inhibit mesangial expansion by inhibiting the TGF-*β* signaling pathway [34]. In addition, klotho might compensate for fetal growth restriction by inhibiting IGF-1 receptor activity in the placenta [21]. Inhibition of IGF-1-PI3K-Akt/PKB-mTOR will lead to mitochondrial dysfunction and IR [35]. The upregulation of protein expression along the IRS-1/PI3K/Akt pathway could lead to an IR decrease [36].

Our study found that the expression of IGF-1R, p-IGF-1R, and IGF-1 was significantly increased in the HG-treated HTR-8/SVneo cells after silencing klotho. Over-expression of klotho significantly reduced the levels of these proteins. It is consistent with previous studies [37]. We further found that klotho silence significantly increased the expression levels of p-Akt and p-mTOR in trophoblast cells. Still, these results were reversed after klotho overexpression. This was consistent with the fact that klotho could reduce the phosphorylation of Akt to weaken insulin signal transduction [22]. These results suggested that klotho gene knockdown could activate the IGF-1/PI3K/Akt/mTOR signaling pathway in trophoblast cells. The effect of klotho on HG-induced IR in HTR-8/SVneo cells might be achieved by regulating the IGF1/PI3K/Akt/mTOR signaling pathway.

## 5. Conclusion

In a word, the results of this study showed that silencing of klotho reversed the decrease of cell activity induced by HG and alleviated insulin resistance. In addition, these results were consistent with klotho's view that it could inhibit the activation of IGF-1 and PI3K-Akt-mTOR signals to promote IR. This study proved that klotho might regulate trophoblast cells' IR under HG conditions, which indicated that klotho might be a promising target for treating GDM.

## Figures and Tables

**Table 1:** Primer sequences.

Gene	Sequences (5′-3′)
Klotho	F: CAGGCGTGGACTCTTCTATGTT
	R: TGATTTTCAGGTAAAGGAGGGA

*β*-actin	F: ACCCTGAAGTACCCCATCGAG
	R: AGCACAGCCTGGATAGCAAC

## Data Availability

The datasets used to support the findings of this study are available from the corresponding author upon reasonable request.
